# Triaptosis and Cancer: Next Hope?

**DOI:** 10.34133/research.0880

**Published:** 2025-09-09

**Authors:** Zi-Zhan Li, Kan Zhou, Jinmei Wu, Lei-Ming Cao, Guang-Rui Wang, Han-Yue Luo, Bing Liu, Lin-Lin Bu

**Affiliations:** ^1^ State Key Laboratory of Oral & Maxillofacial Reconstruction and Regeneration, Key Laboratory of Oral Biomedicine Ministry of Education, Hubei Key Laboratory of Stomatology, School & Hospital of Stomatology, Wuhan University, Wuhan 430079, China.; ^2^ National Key Laboratory of Agricultural Microbiology, College of Chemistry, College of Life Science and Technology, Huazhong Agricultural University, Wuhan 430070, Hubei, China.; ^3^ Department of Oral & Maxillofacial - Head Neck Oncology, School & Hospital of Stomatology, Wuhan University, Wuhan 430079, China.

## Abstract

Cancer persists as one of the most formidable global public health crises and socioeconomic burdens of our era, compelling the scientific community to develop innovative and diversified therapeutic modalities to revolutionize clinical management and enhance patient outcomes. The recent seminal discovery by Swamynathan et al. has unveiled menadione, a vitamin K precursor, as a potent inducer of triaptosis—a novel regulated cell death pathway mediated through the oxidative modulation of phosphatidylinositol 3-kinase PIK3C3/VPS34. This mechanistically distinct cell death paradigm, characterized by its intimate association with endosomal dysfunction and oxidative stress-induced cellular catastrophe, has demonstrated remarkable therapeutic efficacy in preclinical prostate cancer models, outperforming conventional therapeutic regimens and emerging as a potential paradigm-shifting strategy in oncology. This comprehensive review provides a critical synthesis of the triaptosis discovery landscape, elucidating its molecular intricacies and pathophysiological implications. We systematically examine the multifaceted roles of endosomal biology in oncogenesis and tumor progression, while offering a nuanced perspective on redox homeostasis in malignant cells and the therapeutic potential of oxidative stress modulation. Furthermore, we address the inherent dichotomy of oxidative stress induction in cancer therapy, balancing its therapeutic promise against potential adverse effects. Looking toward the horizon of cancer research, we explore transformative therapeutic strategies leveraging triaptosis induction and its potential applications beyond oncology, aiming to catalyze a new era of precision medicine that ultimately enhances patient survival and quality of life.

## Introduction

Cancer stands as a formidable and pivotal issue of global significance, impacting society, economics, and public health on a monumental scale, accounting for nearly one-sixth of all mortalities worldwide and posing a formidable barrier to the extension of human life expectancy [1]. By the year 2050, the annual incidence of new cancer cases is anticipated to soar to 35 million, marking an increase of 77% compared to the levels observed in 2022 [2]. The sheer magnitude of cancer, coupled with the diversity in its profiles across diverse global regions and levels of human development, underscores with renewed emphasis the imperative for an escalated and targeted approach to cancer control on a global scale [3]. Investments in cancer treatment hold the promise of saving countless lives across the globe and delivering substantial economic and social dividends to nations in the forthcoming decades. The dysregulation and resistance of programmed cell death in cancer cells are pivotal characteristics of cancer progression, serving as important drivers of cancer metastasis [3,4]. Inducing programmed cell death in cancer cells stands out as an innovative and promising therapeutic strategy in the realm of cancer treatment. Additionally, the integration of this induction with other therapeutic modalities has emerged as a potential strategy to address cancers that are refractory or tolerant to conventional treatments, thereby enhancing the overall efficacy of cancer therapy [5].

Immunogenic cell death (ICD) orchestrates the activation of the immune system against host cancers, entailing the liberation of damage-associated molecular patterns (DAMPs) from moribund tumor cells [6,7]. This process ignites tumor-specific immune responses, thereby fostering long-term anticancer drug efficacy through the tandem action of direct cancer cell eradication and antitumor immunity [8]. ICD induction, encompassing phenomena such as pyroptosis, ferroptosis, cuproptosis, and disulfide death, has emerged as a cornerstone mechanism in cancer immunotherapy, exhibiting encouraging therapeutic outcomes [9,10]. Though rarely sufficient as a stand-alone anticancer modality, more often serving as an adjunct or synergistic strategy, ICD remains indispensable in cancer treatment, particularly given its promising potentiation of immunotherapy responses [11]. Immunosuppression is an important cause of poor immunotherapy outcomes in many patients with metastatic cancer. For example, immunosuppression in metastatic lymph nodes has recently been reported to lead to immunotherapy resistance [12,13]. Meanwhile, lymph node metastasis induces systemic immune tolerance, which promotes distant metastasis [14]. Our team summarizes the whole process of lymph node metastasis as the “PUMP+” principle, which emphasizes how lymph node metastasis is a step-by-step transformation process that induces lymph nodes, which are important immune organs, to become murderers promoting systemic immune tolerance to distant metastasis [4]. ICD can help to reverse the immunosuppression in the immune microenvironment of the primary and metastatic foci, which is also known as transforming “cold tumors” into “hot tumors”, thus solving the bottleneck of immunotherapy for a long time [15]. A refined comprehension of additional ICD modalities promises to ameliorate the challenge of cancer treatment resistance, while the interplay among diverse cell death pathways may unveil novel therapeutic targets in cancer management [16,17].

A seminal study, recently featured in *Science*, has unveiled a groundbreaking discovery: menadione, a precursor compound of vitamin K, possesses the capacity to induce glutathione (GSH) depletion and generate reactive oxygen species (ROS) [18]. Through the oxidation of phosphatidylinositol 3-kinase PIK3C3/VPS34, it triggers a unique cell death pathway denominated triaptosis. This oxidative process results in endosomal dysfunction, thereby underscoring the detrimental impact of excessive oxidative stress (OS) on cellular structures and its role in activating specific cell death mechanisms [19]. Intriguingly, the study showcases promising therapeutic efficacy in a mouse model of prostate cancer. Given the indispensable role of endosomal sorting in antigen presentation and immune cell activation, triaptosis emerges as a highly plausible novel form of cell death [20]. This revelation holds immense potential for paving innovative therapeutic pathways in the treatment of diverse cancer types ( Table 1).

**Table 1.** Comparison between several different forms of regulated cell death. “–” means unknown content.

Forms	Morphological features	Biochemical features	Immunological features	Key regulator	
Triaptosis [18]	Cytoplasmic vacuole accumulation, plasma membrane blebbing, and terminal burst of cells	PI(3)P depletion	–	Positive •MTM1 •KEAP1	Negative •NFE2L2
Cuproptosis [98]	Mitochondrial volume decreases, membrane density increases, and even swelling and cavitation in mitochondria	Intracellular copper accumulation, oligomerization of lipoylated TCA cycle proteins, and loss of Fe-S cluster proteins	Release of TAAs and DAMPs	Positive •FDX1 •LIAS •LIPT1 •DLD •DLAT •PHDA1 •PDHB •p53 •AARS1/AARS2 •METTL16	Negative •MTF1 •GLS •CDKN2A •ATOX1 •ABCB7 •SIRT2
Ferroptosis [99]	Rounded morphology; shrunken mitochondria with dense membranes and cristae loss; ruptured outer mitochondrial membrane	MAPK activation by Fe and ROS inhibits system Xc^−^, reduces cystine uptake, depletes GSH, oxidizes NADPH, and triggers arachidonic acid mediator release	Release of DAMPs	Positive •VDAC2/3 •Ras •NOX •TFR1 •p53 •CARS	Negative •GPX4 •SLC7A11 •HSPB1 •NRF2
Disulfidptosis [100]	F-actin cytoskeletal collapse, cell rounding, intact membrane, no nuclear condensation	Aberrant accumulation of intracellular disulfides, glucose starvation, aberrant disulfide bonds in actin cytoskeleton proteins, F-actin collapse	–	Positive •SLC7A11 •Rac	Negative •WRC

DAMPs, damage-associated molecular patterns; GSH, glutathione; NADPH, nicotinamide adenine dinucleotide phosphate hydrogen; PI(3)P, phosphatidylinositol 3-phosphate; TAAs, tumor-associated antigens; TCA, tricarboxylic acid cycle

This review offers a comprehensive and insightful examination of the discovery trajectory and underlying mechanisms of triaptosis, while concurrently elucidating the profound implications of OS balance and its perturbations in the progression of cancer. Beyond this, we explore the pivotal roles that the induction of OS and resistance to it play in cancer treatment. Moreover, we offer a forward-looking perspective on future research endeavors in triaptosis and present compelling prospects for the formulation of anticancer therapies through the induction of triaptosis, along with its promising avenues for therapeutic intervention in a spectrum of diseases.

## Discovery of Triaptosis: A Novel Endosome-Dependent Mode of Cell Death

Antioxidants have long been postulated to forestall cancer by virtue of their capacity to neutralize ROS, thereby preventing DNA damage and mutations [21]. Nonetheless, a multitude of large-scale randomized clinical trials have consistently failed to uphold this hypothesis, casting doubt on the universal applicability of antioxidants in cancer prevention. In stark contrast, pro-oxidants have emerged as potential therapeutic agents with promising prospects. Swamynathan et al. made a groundbreaking discovery, revealing that menadione possesses the remarkable ability to induce oxidative cell death. When added to drinking water at a concentration of 0.15 mg/ml, menadione significantly slowed the progression of prostate cancer in genetically engineered mouse models for an extended period of over 18 weeks. This innovative approach demonstrated favorable tolerability and had no adverse impact on coagulation. Furthermore, it provided a more prolonged antitumor effect when compared to androgen deprivation therapy, which is currently the frontline treatment for symptomatic metastatic prostate cancer.

To delve deeper into the mechanisms underlying menadione’s antitumor activity, further in vitro experiments were conducted across a diverse panel of 100 cancer cell lines. Intriguingly, the results revealed that cells harboring mutations or depletion of Kelch-like ECH-associated protein 1 (KEAP1)—a key regulator that stabilizes the NFE2-like basic leucine zipper transcription factor 2 (NFE2L2) and enhances the antioxidant response—exhibited increased resistance to menadione. These findings underscore the complex interplay between redox biology and therapeutic efficacy, suggesting that the intrinsic redox buffering capacity of cancer cells may influence their responsiveness to pro-oxidant therapies.

**Fig. 1.**
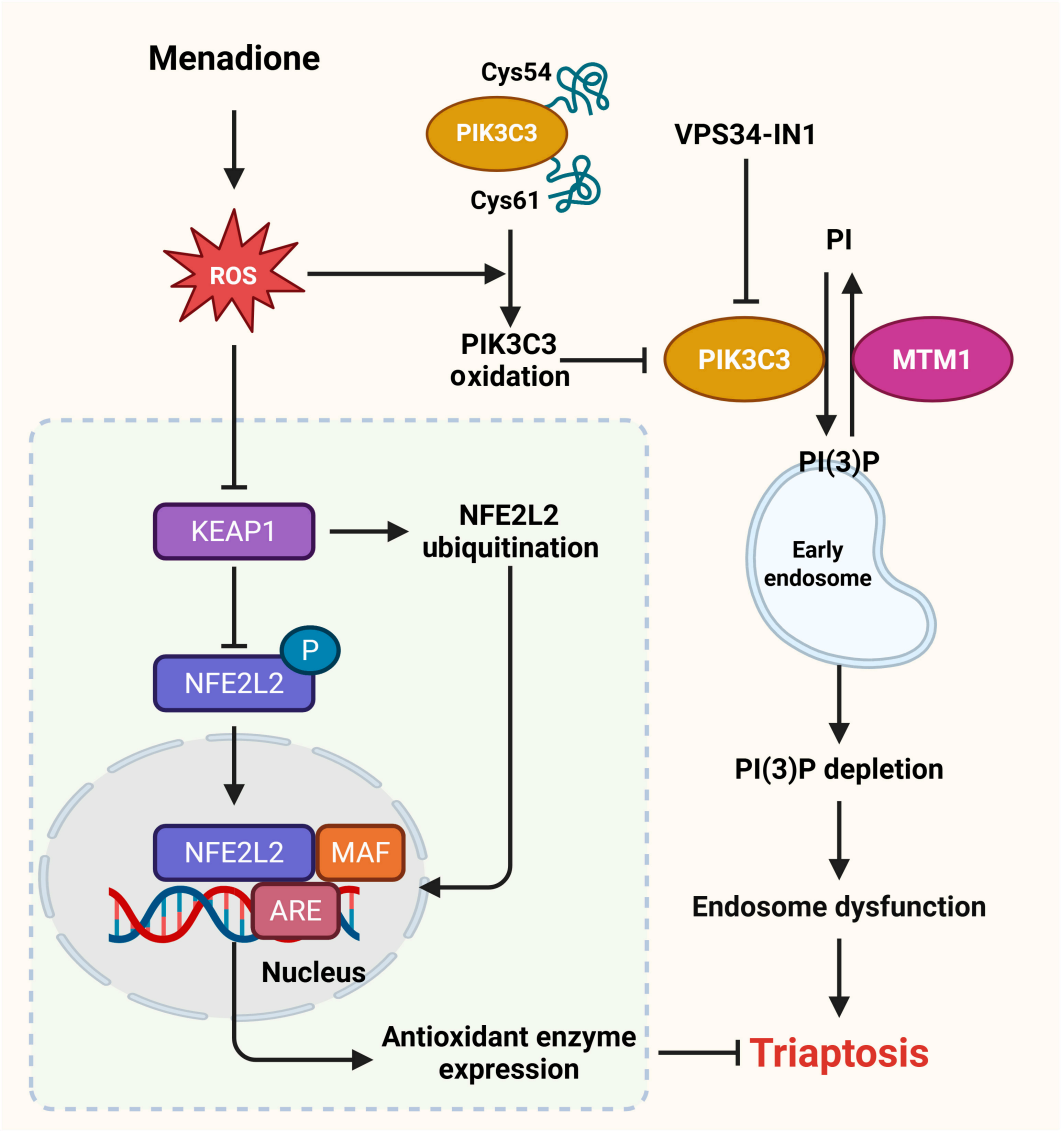
The mechanism of triaptosis. Mendione acts as a pro-oxidant inducing intracellular production of ROS, which leads to the oxidation of the cysteine residues of PIK3C3. PIK3C3 carries out most of the synthesis of PI(3)P, and oxidation leads to the depletion of PI(3)P in the endosomes, which, in turn, leads to endosomal dysfunction, and ultimately to triaptosis. In addition, excess intracellular ROS interfered with KEAP1-mediated ubiquitin-proteasome degradation of NFE2L2, which activated NFE2L2. Subsequently, NFE2L2 undergoes nuclear translocation and binds to antioxidant response elements (AREs) and drives the expression of genes for antioxidant stress enzymes.

Despite the transcriptome alterations induced by menadione bearing a striking resemblance to those elicited by ferroptosis inducers, no standard cell death inhibitors were found to mitigate menadione’s cytotoxic effects. This observation hinted at the possibility that menadione may trigger a unique oxidative cell death pathway distinct from ferroptosis. Morphological analysis provided further insights into the mode of action of menadione. The presence of substantial cytosolic vacuole accumulation in menadione-treated cells suggested that it may disrupt endosomal function, leading to membrane blistering and rupture. To substantiate this hypothesis, gene set enrichment analysis was performed, revealing that blocking the early stages of endocytosis and endosome formation sensitized cells to menadione, whereas inhibiting late endosome (LE) progression diminished its therapeutic efficacy. Importantly, menadione was identified as a direct target of phosphatidylinositol 3-kinase catalytic subunit type 3 (PIK3C3, also known as VPS34), a class III phosphoinositide 3-kinase crucial for endosome formation and intracellular sorting. Swamynathan’s study has clearly demonstrated that menadione induces site-specific, reversible oxidation of PIK3C3 (VPS34), particularly at Cys54 and Cys61 within the C2 domain. These cysteines are essential for kinase function, and their oxidation leads to VPS34 complex inactivation and downstream phosphatidylinositol 3-phosphate [PI(3)P, a defining lipid of early endosomes (EE)] depletion. Mutagenesis and rescue experiments confirmed that menadione-induced oxidative cell death is mediated, at least in part, through this redox-sensitive regulatory switch. This depletion of PI(3)P caused endosomal dysfunction in prostate cancer cells, ultimately resulting in membrane blistering, rupture, and the induction of a unique oxidative cell death pathway termed triaptosis (Fig. 1) [18].

## The Pivotal Role of Endosomes in Cancer Cells

Endosomes, membrane-encapsulated organelles, serve as transient vesicular carriers in the trafficking of materials across eukaryotic cellular boundaries, orchestrating fundamental processes encompassing nutrient assimilation, immune responses, signal relay, adhesion dynamics, membrane turnover, and ontogenesis [22,23]. Components internalized via diverse endocytic routes converge into a unified EE compartment [24]. Clathrin-mediated endocytosis (CME) is a constitutive endocytosis pathway common to all cells. Phosphoinositol, a signaling lipid derived from phosphatidylinositol (PI), directs inward and outward membrane flow in a variety of endocytosis and extracellular binding pathways. During CME, phosphatidylinositol 4,5-bisphosphate [PI(4,5)P_2_] promotes the nucleation and growth of endocytosed clathrin-coated pits (CCPs). The maturing CCP acquires a variety of phosphatidylinositol metabolizing enzymes such as PIK3C2α, which lead to the gradual conversion of PI(4,5)P_2_ to phosphatidylinositol 3,4-bisphosphate [PI(3,4)P_2_]. The further effect of PI(3,4)P_2_ results in the contraction of the neck of the CCP, which is a precondition for the cleavage of the endocytosed vesicle. Subsequently, the newly formed vesicles recruit the cochaperone protein auxilin or GAK to trigger removal of the clathrin coat. PI(3,4)P_2_ is then converted by endosomal 4-phosphatase to PI(3)P, which ultimately fuses with the EE. PI(3)P is the signature lipid of the EE and determines sorting of endocytosed cargo. Endocytosed cargo can be recycled to the plasma membrane, retrogradely transported to the *trans*-Golgi network or sorted into LE for lysosomal degradation [25]. The progression from early to LE entails extensive protein and lipid remodeling and alterations in the luminal milieu of endosomes, with LE functioning as a secondary trafficking hub and sorting nexus within the endosomal network [26].

**Fig. 2.**
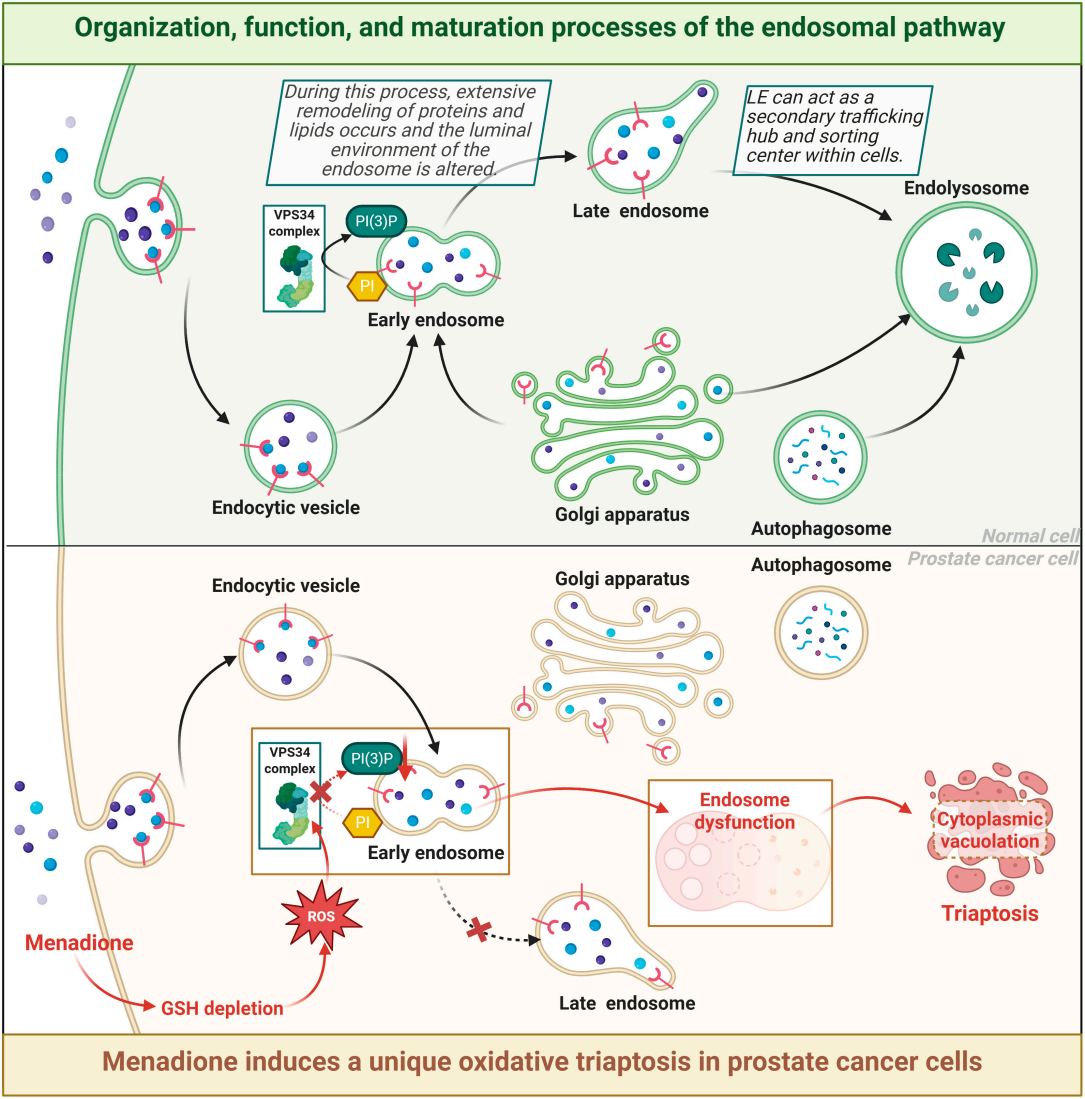
Organization, function, and maturation processes of the intracellular endosomal pathway, and the role of endosomes in triaptosis. Cargoes endocytosed via different endocytosis pathways converge into a unified early endosome. Early endosomes undergo protein and lipid remodeling as well as changes in the environment within the intronic lumen to transform into late endosomes. Late endosomes function as secondary transport hubs and sorting centers in the endosomal network. The depletion of PI(3)P caused by ROS then leads to an early endosomal inability to properly sort cargo, which eventually builds up into huge vacuoles, causing the cell to swell and rupture toward death.

In the study by Swamynathan, leveraging mechanistic insights from a comprehensive genome-wide CRISPR screen in the human metastatic PC3 cell line, coupled with bioinformatic analyses, unveiled that interfering with the initial stages of endocytosis and endosome formation rendered cells susceptible to menadione, whereas impeding LE maturation mitigated its therapeutic impact. Intriguingly, PI(3)P is synthesized from PIs mainly via VPS34, with a minor contribution from class II phosphatidylinositol 3-kinase. Thus, when VPS34 oxidation leads to a decrease in PI(3)P levels, the alternatives, class I and II phosphatidylinositol 3-kinases and multiple PIP metabolizing enzymes, are unable to prevent triaptosis following inactivation of the VPS34. In contrast, however, cells with sufficient reducing reserves can overcome this stress by completely rescuing triaptosis through thiol-reducing capacity (Fig. 2) [18,20].

Indeed, the biology of endosomes is instrumental in the ontogeny and progression of cancer. During asymmetric stem cell division, perturbations in endocytosis and endosomal function can unleash unregulated receptor tyrosine kinase and G protein-coupled receptor signaling cascades, precipitating exponential cellular proliferation—a hallmark of oncogenic transformation [27,28]. This process may be intertwined with deregulation of Notch signaling, which further exacerbates the imbalance in endosomal sorting [29]. In *Drosophila* neuroblasts, asymmetric activation of Notch fosters self-renewal and inhibits differentiation in 1 of the 2 progeny cells, ensuring one neuroblast persists while the other undergoes differentiation. Notably, unchecked differentiation potential could precipitate tumorigenesis [30]. Moreover, alterations in the expression profiles of endosome-associated proteins in diverse cancer types mirror cancer progression trends [31]. Overexpression of LIMP-2 is associated with augmented endosomal biogenesis and expansion of both EE and LE compartments [32]. Enhanced expression and aberrant peripheral distribution of EE may promote heightened nutrient acquisition, increased membrane delivery, and aberrant intracellular signaling, which have been implicated in the progression of pancreatic cancer (PC) and oral squamous cell carcinoma (OSCC) [33–35]. Furthermore, specific patterns of endosomal gene expression may serve as prognosticators for patient outcomes. In PC, overexpression of EE-related genes, namely, APPL1, EEA1, and RAB5A, correlates with aggressive disease phenotypes and poorer clinical prognoses [36]. These findings underscore the sophisticated interplay between endosomal biology and oncogenic processes, hinting at novel therapeutic strategies targeting endosomal dysfunction in cancer treatment [37].

## OS in Cancer

In the cascade leading to triaptosis, menadione triggers GSH depletion and the generation of ROS, resulting in the oxidation of specific cysteine residues (Cys54 and Cys61) on PIK3C3. This oxidative modification constitutes a critical antecedent to endosomal dysfunction in PC cells, ultimately culminating in triaptosis, with GSH depletion serving as an indispensable determinant of menadione’s cytotoxic efficacy [18]. Morphological examination provides compelling evidence that triaptosis constitutes an oxidative cell death pathway, characterized by prominent cytoplasmic vacuolation in PC cells. Considering GSH’s multifaceted participation in diverse cell death paradigms, including apoptosis, necroptosis, and ferroptosis, where it plays indispensable roles, there are intriguing convergences among various OS-linked cell death modalities, including triaptosis [38]. These convergences have garnered extensive attention in the realm of cancer biology research, highlighting their potential as key targets for therapeutic intervention.

### Maintenance of redox balance and OS homeostasis in cancer

In the context of normal cellular physiology, the generation of ROS and reactive nitrogen species (RNS) represents an inevitable outcome of metabolic processes [39]. To ensure the preservation of ROS/RNS signaling while mitigating oxidative damage, cells harbor an intricate array of antioxidant systems [40]. Beyond directly acting antioxidants, cells possess indirectly acting antioxidant mechanisms that restrict the formation of ROS/RNS or detoxify the reactive metabolites they produce [41]. An imbalance characterized by a disproportionate increase in ROS/RNS relative to antioxidant capacity is defined as OS, which cells counteract through multifaceted strategies [42]. Notably, GSH and thioredoxin (TXN) occupy central stages in mitigating OS, with their functional capacities sustained by NADPH, which maintains both antioxidants in their reduced states [43].

In the intricate landscape of cancer progression, ROS exerts a paradoxical influence on cancer evolution. On the one hand, it can initiate or stimulate tumorigenesis, fostering cancer cell transformation and proliferation [44]. On the other hand, it can induce cell death. The hyperproliferation of cancer cells is often accompanied by heightened ROS production, which is attributed to oncogene-induced and/or damage-stimulated mitochondrial peroxide generation [45]. Remarkably, tumor cells adapt to thrive under conditions where this oxidative burden shifts the redox balance away from a reduced state. They accomplish this by augmenting their antioxidant status to optimize ROS-driven proliferation while circumventing the ROS threshold that triggers senescence and oxidative cell death [46]. Consequently, elevated levels of GSH, TXN, and/or thioredoxin reductase are frequently observed in various cancer types, potentially as a defensive mechanism against high ROS loads, which correlates significantly with adverse patient prognosis [47]. Beyond bolstering the antioxidant system to alleviate OS, tumor cells can also stimulate anti-apoptotic and pro-survival pathways [48]. For example, breast cancer cells exploit redox-sensitive transient receptor potential ankyrin 1 channels to activate Ca^2+^ signaling, thereby stimulating the extracellular regulated protein kinases and phosphoinositide-3-kinase–protein kinase B pathways [49]. This, in turn, activates myeloid cell leukemia-1, leading to OS tolerance and drug resistance, demonstrating the sophisticated mechanisms employed by cancer cells to survive and proliferate in the face of oxidative challenges.

### Harnessing OS for cancer therapy

Induction of OS in tumor cells has long been used to achieve tumor suppression. This strategy often involves locally targeted delivery of oxidants to tumors using drug delivery systems (DDSs), including through photodynamic therapy (PDT) [50], sonodynamic therapy (SDT) [51], and a variety of metal-based nanoenzymes [52]. However, pro-oxidant therapy specifically induces triaptosis in tumor cells by increasing systemic OS and targeting the weakness of PI(3)P deficiency in tumor cells [53]. Of course, pro-oxidant therapy is also a double-edged sword, which raises OS throughout the body and can have some side effects while treating cancer.

#### Targeting pro-oxidants for cancer therapy

Pro-oxidant therapy holds promise in retarding cancer progression, potentially via exacerbating OS in cancer cells or disrupting metabolic adaptations that confer resistance to OS [20,54]. Encouragingly, several studies suggest that cancer cells are more susceptible to OS-induced damage than normal cells, fostering optimism that pro-oxidant therapy may emerge as a pivotal hope in cancer treatment [55]. This therapy can be realized by either directly generating ROS or indirectly elevating intracellular ROS concentrations by targeting and inhibiting cancer cells’ endogenous antioxidant systems [56]. Indeed, numerous Food and Drug Administration-approved anticancer drugs effectively eliminate cancer cells by augmenting ROS production [57]. For instance, chemotherapeutic agents that interfere with cell division, such as vinca alkaloids and taxanes, or those that disrupt nucleic acid synthesis, like 5-fluorouracil, platinum compounds, and anthracyclines, can trigger ROS generation by disrupting mitochondrial function [57]. The specific chemical structure of anthracyclines allows them to be reduced to semiquinone forms. This process is catalyzed by NADPH oxidase (NOX) and nitric oxide synthase in the cytoplasm, as well as the mitochondrial electron transport chain (ETC) [58,59]. These components transfer electrons to doxorubicin, forming semiquinone complexes, which are unstable metabolites that can be oxidized by oxygen in mitochondria, accompanied by the release of substantial ROS [37,60]. In recent years, exciting preclinical results have been reported for novel drugs (small-molecule compounds) that increase intracellular ROS levels. Elesclomol (STA-4783), a synthetic small-molecule compound, exhibits anticancer activity in various cancers, such as melanoma and breast cancer. Elesclomol chelates copper ions and transports them into mitochondria, disrupting the mitochondrial respiratory chain and augmenting cellular OS, a process termed cuproptosis, ultimately leading to cancer cell apoptosis [60,61]. Several clinical trials (NCT00808418, NCT00088088, and NCT00087997) have been conducted to evaluate the safety and efficacy of elesclomol, either alone or in combination with conventional chemotherapy drugs, for treating various cancers. Beyond direct ROS inducers, pro-oxidant strategies targeting intracellular antioxidant systems and disrupting their balance can also elicit OS [6]. Antioxidant processes involve ROS neutralization and disulfide reduction, relying on the regulation of 2 metabolic pathways: GSH and thioredoxin (TRX) [62,63]. Additionally, PDT and SDT are modern, noninvasive treatments for cancer and non-neoplastic diseases. By exciting photosensitizer molecules, PDT generates toxic ROS using absorbed light under near-infrared laser irradiation. Similarly, SDT employs ultrasound to activate sonosensitizer drugs, which then produce ROS and kill cancer cells [64,65]. Currently, numerous clinical and preclinical studies of PDT in various stages have been initiated (NCT03638622, NCT03527225, and NCT01012401).

Furthermore, ascorbic acid (vitamin C), commonly regarded as an antioxidant, can reach super-physiological levels through intravenous infusion, leading to the uptake of its fully oxidized form, dehydroascorbic acid, via GLUT1 transporters highly expressed in cancer cells with activated mitogen-activated protein kinase (MAPK) pathways [66,67]. Once absorbed by cancer cells, dehydroascorbic acid is reduced back to ascorbic acid, inducing OS by consuming reducing equivalents. Multiple clinical studies have reported extended survival in cancer patients receiving high-dose intravenous ascorbic acid [68]. Dietary interventions may also exert pro-oxidant effects. The ketogenic diet, which increases dietary fat intake, typically elevates polyunsaturated fatty acid (PUFA) levels [69]. Increased incorporation of PUFAs into membrane phospholipids renders cancer cells more susceptible to lipid ROS accumulation and ferroptosis [70].

Pro-oxidant therapy as a novel anticancer mechanism may have a role in the treatment of metastatic cancer. On the one hand, a large number of studies have confirmed that antioxidants act as metastasis promoters during cancer metastasis [71]. Compared to primary tumors, cancer cells experience higher OS during metastasis and rely on NADPH to maintain redox homeostasis. Folic acid supplementation, as an antioxidant, helps to promote NADPH production through the folate pathway, thus promoting metastasis [72]. On the other hand, it has been demonstrated that NRF2 plays a key role in lymph node metastasis of cancer [73]. NRF2 reverses the decrease in free heme levels caused by oral antioxidants, which, in turn, elevates the level of BACH1 protein and promotes the development of lung metastasis [74]. In a study by Swamynathan et al., they utilized an oral pro-oxidant against the KEAP1-NRF2 antioxidant system, successfully induced triaptosis in PC cells, thereby effectively inhibiting the progression of metastatic PC. In addition, it has been suggested that the lymph nodes are a haven for metastatic cancer cells to escape OS and that exposure to the lymphatic environment also increases the ability of cancer cells to survive in the bloodstream [75]. Thus, the blind spot for pro-oxidant therapy may exist in the lymphatic system. Perhaps pro-oxidant therapy by targeting metastatic lymph nodes could better compensate for this and inhibit systemic metastasis to some extent.

Although many pro-oxidant-based anticancer therapies demonstrate promising effects, the extent to which their anticancer activity reflects these pro-oxidant activities, as opposed to other ROS-independent activities, remains uncertain and necessitates deeper mechanistic understanding and validation through large cohort clinical trials.

#### The role of OS in cancer therapy: A double-edged sword

Despite exhibiting promising antitumor efficacy and achieving partial advancements in clinical trials, pro-oxidants and other agents that elicit OS present a concerning array of potential adverse effects stemming from their inherent lack of targeting specificity during systemic circulation [76,77]. Specifically, these agents demonstrate a heightened affinity for rapidly proliferating tissues, including the gastrointestinal tract, bone marrow, and hair follicles [78]. Furthermore, the toxicity induced by OS in vital organs such as the heart, liver, lungs, kidneys, and gastrointestinal tract markedly undermines their clinical utility [79].

Anthracyclines, a prominent class of chemotherapy agents, exemplify the adverse cardiac effects associated with OS, thereby catalyzing the emergence and evolution of the specialized discipline of cardio-oncology [80,81]. A primary contributor to anthracycline-induced cardiomyopathy is the mitochondrial DNA damage mediated by OS. Enzymatic reduction of anthracyclines to semiquinones, followed by reaction with oxygen, generates superoxide anions (O^2−^), which are subsequently converted into H_2_O_2_ by superoxide dismutase [82]. While H_2_O_2_ is relatively stable and low in toxicity, it can undergo iron-catalyzed Fenton reactions with O^2−^ to produce highly reactive and toxic hydroxyl radicals (•OH) [83]. This excessive production of ROS damages multiple mitochondrial components, disrupts normal mitochondrial function, and reduces energy production efficiency. Given that the myocardium consumes the highest amount of energy per gram of tissue, with mitochondria comprising one-third of the cell’s total volume, mitochondrial dysfunction profoundly impacts the myocardium, which relies on high metabolic demands and energy consumption [84,85]. Additionally, pro-oxidant-induced OS may contribute to “chemo brain” symptoms, with mitochondrial dysfunction [86], elevated levels of tumor necrosis factor-α, and the translational effects of the drugs themselves serving as potential sources of high ROS [87]. Despite the protective mechanisms of the blood–brain barrier (BBB), ROS can disrupt and downregulate the BBB through downstream cytokine-mediated signaling events, ultimately leading to cognitive impairment and peripheral neuropathy [88]. The liver, as the primary metabolic site for most chemotherapy drugs, generates substantial ROS during the detoxification of exogenous and toxic substances [89]. Associated OS has been implicated in liver diseases, such as hepatotoxicity, and other pathological conditions. Drug-induced hepatic OS can lead to lipid peroxidation, mitochondrial dysfunction, endoplasmic reticulum stress, and DNA damage, directly causing drug-induced liver injury, which is a major reason for drug discontinuation in preclinical and clinical stages [90,91].

**Fig. 3.**
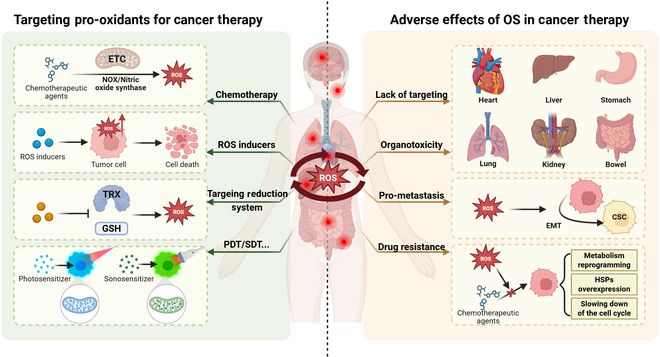
Various pathways of targeted pro-oxidants for tumor therapy and potential side effects. Some chemotherapeutic agents can induce large amounts of ROS in the cytoplasm catalyzed by ETC and NADPH oxidase (NOX) and nitric oxide synthase. Targeting inhibition of intracellular reduction systems can also enhance oxidative stress (OS). In addition, exogenous ROS can be introduced by ROS inducers and through photodynamic therapy (PDT) and sonodynamic therapy (SDT). However, pro-oxidant therapies often cause elevated levels of OS in systemic organs and tissues due to a lack of targeting, and thus may be accompanied by some organ toxicity. Intratumoral OS levels promote EMT transformation of tumor cells, thereby promoting metastasis. Moreover, OS therapy may bring about drug resistance.

Notably, pro-oxidant-induced OS is not solely a therapeutic strategy for cancer but may also become a crucial factor impairing anticancer efficacy. Some pro-oxidant therapies can promote ROS-induced drug resistance [92]. Possible mechanisms include lipid peroxidation and lipid metabolism reprogramming during chemotherapy drug-induced OS, generating a large amount of electrophilic aldehydes that can slow down the cell cycle of cancer cells, thereby reducing the efficacy of drugs that rely on blocking the cell replication cycle to induce cancer cell death [93]. Furthermore, heat shock proteins, which serve as molecular chaperones with potent protective effects on cells, may be overexpressed during acute or chronic OS, leading to protein misfolding, protein aggregation, and regulatory complex disruption [94]. This may result in reduced cancer cell apoptosis and confer resistance to OS-based chemotherapy. Additionally, OS may alter cellular phenotypes, with ROS promoting epithelial–mesenchymal transition (EMT), thereby conferring cancer stem cell-like characteristics to cancer cells, such as high self-renewal and drug/radiation resistance [95]. Enhanced ROS scavenging capacity, manifested as enhanced mitochondrial respiration and GSH production, helps cancer cells maintain low ROS levels or even a quiescent state to evade damage during pro-oxidant therapy (Fig. 3) [96].

## Conclusion and Prospects

Triaptosis has emerged as a groundbreaking endosome-dependent, OS-mediated programmed cell death mechanism, representing a paradigm shift in understanding of oncological cell fate regulation. Mechanistically, pharmacological perturbation of EE biogenesis potentiates malignant cells’ vulnerability to pro-oxidant agents, whereas disruption of late endosomal maturation cascades confers resistance, mirroring the spatiotemporal dynamics of triaptosis pathway activation. The therapeutic potential of OS induction in oncology, despite the robust redox homeostasis machinery inherent in neoplastic cells, has garnered substantial attention in translational research. Nevertheless, the “double-edged sword” effect nature of pro-oxidant therapeutics necessitates meticulous evaluation of their therapeutic index in clinical oncology practice. This seminal discovery of triaptosis not only expands the molecular armamentarium against cancer but also heralds a new era in targeted oncotherapy. We envision that the clinical translation of triaptosis-centric therapeutic modalities will revolutionize precision medicine paradigms, ultimately enhancing therapeutic outcomes and quality of life for cancer patients.

In the investigation conducted by Swamynathan et al., menadione was unequivocally demonstrated to orchestrate triaptosis through the orchestrated depletion of GSH and concomitant generation of ROS, with GSH depletion constituting an indispensable molecular prerequisite for this regulated cell death (RCD) paradigm. Nevertheless, the mechanistic intricacies underlying triaptosis remain enigmatic, necessitating comprehensive and multifaceted investigations to delineate its molecular circuitry and harness its therapeutic potential in oncological interventions. Intriguingly, a panoply of cell death modalities, including apoptosis, autophagy, ferroptosis, and cuproptosis, exhibit convergent features of GSH depletion and ROS accrual, intimating potential mechanistic interconnections at the nexus of these pathways. The advent of cutting-edge multi-omics approaches and sophisticated bioinformatics has catalyzed a paradigm shift in our comprehension of cell death mechanisms and resistance landscapes. We must acknowledge the pioneering contributions of Swamynathan et al. while recognizing that all studies have limitations. Their investigation into triaptosis mechanisms was not exhaustive. Although they established that NFE2L2 enhances menadione resistance, the underlying molecular mechanisms and downstream targets remain unidentified. Additionally, while Swamynathan et al. characterized triaptosis as an OS-dependent cell death process, they did not investigate whether it is immunogenic. This knowledge gap hinders the development of triaptosis-based therapeutics and requires further experimental validation.

**Fig. 4.**
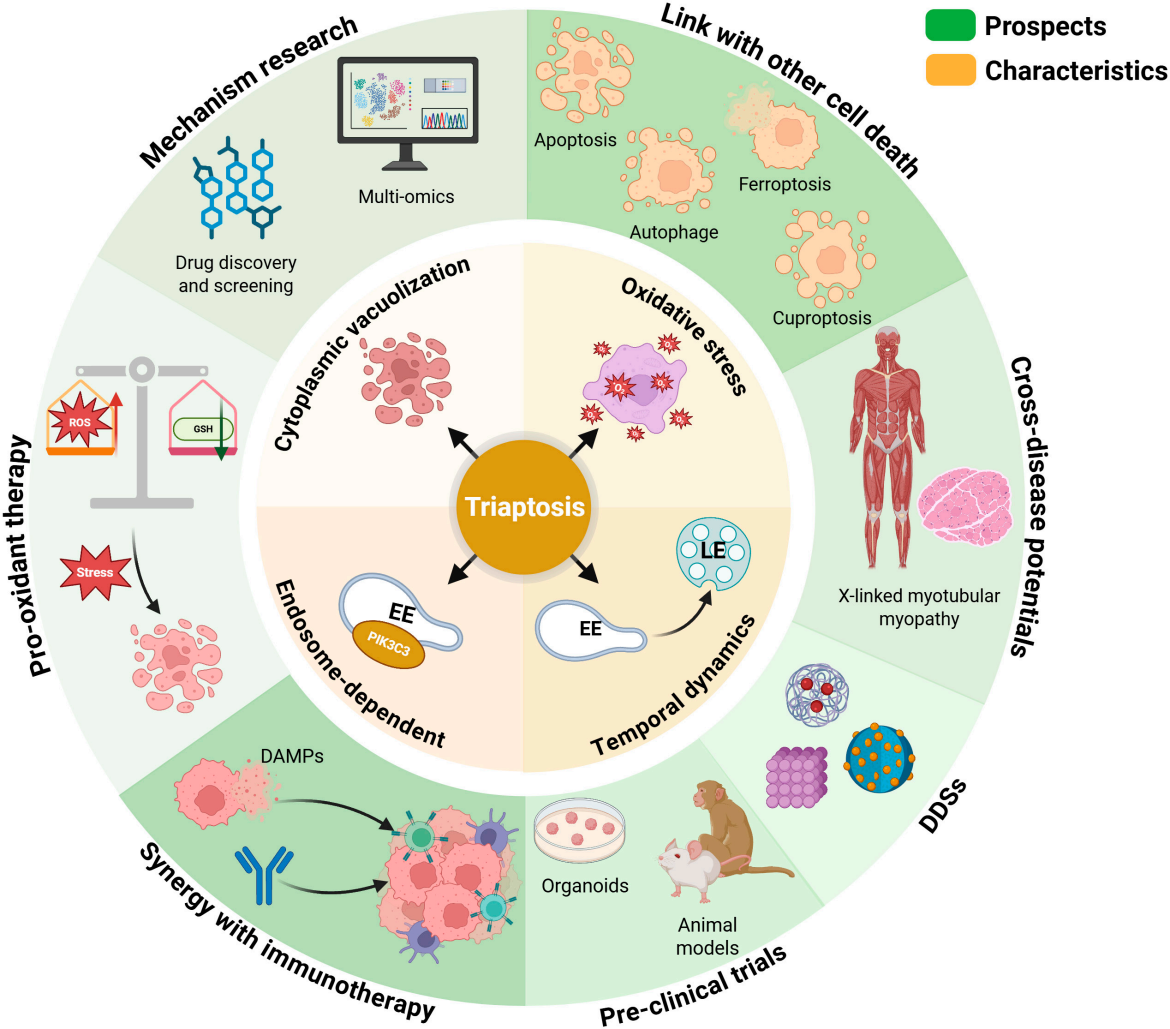
Four key characteristics and future prospects of triaptosis. According to the summary of available studies, there are 4 key features of triaptosis, including cytoplasmic vacuolization, induction of OS, dependence on endosomes, and temporal dynamics. Triaptosis holds the promise of being a novel therapeutic strategy for tumors, and its mechanisms should be further explored and the links between it and other cell deaths should be developed. In addition, in order to exploit its therapeutic potential, its synergy with immunotherapy could be explored and precisely targeted drug delivery systems (DDSs) based on triaptosis could be developed. More drugs mediating pro-oxidant therapies and triaptosis should also be developed and screened as well as advancing preclinical experiments and clinical studies of the drugs. Finally, the therapeutic potential of triaptosis in diseases other than tumor remains to be explored.

Recent years have witnessed an unprecedented surge in therapeutic modalities targeting the induction of cell death, with small-molecule entities and nano-prodrug architectures demonstrating remarkable preclinical efficacy. However, notwithstanding the progression of select agents to clinical trials, monotherapeutic strategies predicated on cell death induction have manifested limited clinical utility. Consequently, contemporary research endeavors have pivoted toward exploring cell death induction as an adjuvant strategy to potentiate immunotherapy efficacy or circumvent chemoresistance. While triaptosis has not been formally classified as ICD, the pivotal role of endosomes in antigen presentation and immune activation portends its potential to augment immunotherapeutic outcomes. Augmenting the therapeutic index of cell death-inducing agents remains a formidable challenge, particularly in light of the “double-edged sword” nature of pro-oxidant therapies. The dynamic and evolving redox regulatory capacity of cancer cells, often mediated through metabolic reprogramming, poses additional impediments to therapeutic efficacy. Combinatorial approaches targeting both OS-dependent cell death and cancer cell metabolism may herald a promising therapeutic paradigm. Although current evidence supports triaptosis as the primary form of RCD induced by menadione, its strong pro-oxidative nature suggests that menadione treatment likely elevates intracellular OS to levels that may also favor the activation of other RCD pathways. For instance, ferroptosis and cuproptosis are both driven by increased ROS or redox imbalance. Notably, triaptosis shares several features with ferroptosis, including thiol depletion and KEAP1 dependency. Therefore, combining triaptosis with ferroptosis may represent a promising future strategy for OS-based cancer therapies. The development of DDS for the targeted induction of triaptosis represents another innovative strategy [97]. These systems, leveraging chemical modifications or biomimetic principles, enable tumor-specific delivery, potentially achieving “precision strike” effects while mitigating off-target toxicity [17]. This approach holds particular promise for metastatic disease, where DDS could facilitate multifocal targeting and therapeutic synergy (Fig. 4).

Notably, Swamynathan et al. reported that menadione demonstrated therapeutic efficacy in X-linked myotubular myopathy caused by myotubularin 1 deficiency, suggesting therapeutic potential beyond oncology. While the mechanism appears distinct from triaptosis induction, further investigation into this phenomenon is warranted. As we continue to unravel the complexities of this novel cell death modality, triaptosis-based therapeutic strategies may emerge as the “next hope” in cancer therapy, potentially transforming clinical management and improving patient outcomes.
